# Effect of Internet-Based Rehabilitation Programs on Improvement of Pain and Physical Function in Patients with Knee Osteoarthritis: Systematic Review and Meta-analysis of Randomized Controlled Trials

**DOI:** 10.2196/21542

**Published:** 2021-01-05

**Authors:** Su-Hang Xie, Qian Wang, Li-Qiong Wang, Lin Wang, Kang-Ping Song, Cheng-Qi He

**Affiliations:** 1 School of Rehabilitation Sciences West China School of Medicine Sichuan University Chengdu, Sichuan China; 2 Rehabilitation Medicine Center West China Hospital Sichuan University Chengdu, Sichuan China; 3 Key Laboratory of Rehabilitation Medicine in Sichuan Province Chengdu, Sichuan China

**Keywords:** internet-based rehabilitation, knee, osteoarthritis, pain, physical function, meta-analysis, review, telerehabilitation, eHealth, telemedicine

## Abstract

**Background:**

Osteoarthritis (OA) is a chronic, debilitating, and degenerative joint disease. However, it is difficult for patients with knee OA to access conventional rehabilitation when discharging from the hospital. Internet-based rehabilitation is one of the promising telemedicine strategies to provide a means combining monitoring, guidance, and treatment for patients with knee OA.

**Objective:**

The aim of this study was to conduct a systematic review and meta-analysis for assessing the effect of internet-based rehabilitation programs on pain and physical function in patients with knee OA.

**Methods:**

Keywords related to knee OA and internet-based rehabilitation were systematically searched in the Web of Science, MEDLINE, EMBASE, CENTRAL, Scopus, PEDro (Physiotherapy Evidence Database), CNKI, SinoMed, and WANFANG databases from January 2000 to April 2020. Only randomized controlled trials were included. The authors independently screened the literature. The main outcome measures were focused on pain and physical function. A meta-analysis was performed on the collected data. Review Manager (RevMan, version 5.3) was used for all analyses.

**Results:**

The systematic review identified 6 randomized controlled trials, 4 of which were included in the meta-analysis, comprising a total of 791 patients with knee OA. The meta-analysis with the fixed-effects model showed that the internet-based rehabilitation programs could significantly alleviate the osteoarthritic pain for patients compared with conventional rehabilitation (standardized mean difference [SMD] –0.21, 95% CI  −0.4 to –0.01, *P*=.04). No significant difference was found in the improvement of physical function in patients with knee OA compared with conventional rehabilitation within 2 to 12 months (SMD –0.08, 95% CI −0.27 to 0.12, *P*=.43).

**Conclusions:**

This systematic review shows that internet-based rehabilitation programs could improve the pain but not physical function for patients with knee OA. However, there was a very small number of studies that could be included in the review and meta-analysis. Thus, further studies with large sample sizes are warranted to promote the effectiveness of internet-based rehabilitation and to develop its personalized design.

## Introduction

Osteoarthritis (OA) is a chronic, debilitating, and degenerative joint disease, which is widely considered as a significant threat to healthy aging [1-3]. A recent estimation revealed that approximately 250 million individuals are suffering from OA worldwide, and the knee joint is the most frequently affected joint with an incidence of 16%-17% among people aged 50 to 75 years [4,5]. Chronic pain and impaired physical function are recognized as the main issues affecting quality of life for patients with knee OA [6,7]. Physical therapy is one of the effective methods that is commonly prescribed for patients with knee OA to alleviate pain and improve physical function [8-10]. However, it is difficult for patients with knee OA, who are mainly middle-aged to elderly, to access conventional rehabilitation programs that are monitored and guided by physicians or therapists when discharging from the hospital [4]. Thus, it is necessary to develop telerehabilitation strategies to provide the opportunity to access professional rehabilitation programs and guides for improving the long-term outcomes of pain and physical function for patients with knee OA.

The advent of telemedicine has facilitated the access of patients to real-time communication with professional physicians or therapists [11-13]. Telerehabilitation has been developed in the fields of physical medicine and rehabilitation to support continuous rehabilitation services for patients with disabilities [14]. Several types of telerehabilitation services have been proposed, including video conference, telephone conference, and web-based knowledge platforms [15-17]. Of these, internet-based rehabilitation, which combines internet technologies with physical medicine and rehabilitation, could vastly promote accessibility to professional physicians or therapists for patients, even for those residing in remote areas [18,19]. The feasibility of internet-based rehabilitation and its effect have been investigated in patients with stroke [20,21], chronic obstructive pulmonary disease [22-25], Parkinson disease [26-28], multiple sclerosis [29,30] and following knee arthroplasty [31,32]. 

Despite the increasing popularity of these internet-based rehabilitation programs, there is insufficient evidence to demonstrate their effectiveness for patients with knee OA. Positive results have been shown in some studies in which specific programs of internet-based rehabilitation could improve pain and physical function for patients with knee OA as compared with conventional rehabilitation [33,34]. However, different views have also been put forth, indicating no significant change in OA-related pain and physical function during long-term follow up of 12 months [35,36]. To our knowledge, there has been no meta-analysis of randomized controlled trials (RCTs) assessing the effects of internet-based rehabilitation programs on improvement of pain and physical function in patients with knee OA. Therefore, the aim of this systematic review and meta-analysis was to assess the effect of internet-based rehabilitation programs on the pain and physical function of patients with knee OA, and to evaluate the specific components (eg, exercise guidance, knee OA education) designed for each of the internet-based rehabilitation programs reported to date.

## Methods

### Study Protocol and Registration

All analyses were based on data from previously published studies. Thus, no ethical approval or patient consent was required. The review was conducted according to the Preferred Reporting Items for Systematic Reviews and Meta-Analyses (PRISMA) statement [37]. The a priori protocol for the review is published in the International Prospective Register of Systematic Reviews (PROSPERO): CRD42019137907.

### Information Sources

The following electronic databases were searched to identify relevant studies from January 2000 to April 2020: Web of Science, MEDLINE, EMBASE, CENTRAL, Scopus, Physiotherapy Evidence Database (PEDro), CNKI, SinoMed, and WANFANG. Relevant journals, conference proceedings, and reference lists were manually searched to identify additional studies. 

### Search and Eligibility Criteria

#### Overall Search Strategy

The search was performed using a combination of the following keywords on May 1, 2020: (osteoarthritis or osteoarthrosis or cartilage or degenerative arthritis) AND (telemedicine or e-health or telehealth or telerehabilitation or internet or web or online or app or wearable or sensor) AND knee. The search strategies for each database are presented in Multimedia Appendix 1. In addition, the literature was searched manually from the reference lists of the articles identified from the search of the electronic databases. The inclusion and exclusion criteria of the studies were based on the PICO (Population, Intervention, Comparison, Outcome) method [38,39].

#### Studies

RCTs regarding the effect of internet-based rehabilitation programs for patients with knee OA were included in the review. The included studies were published in English or Chinese. Articles were excluded if the study was a non-RCT or nonclinical trial. Abstracts from meeting proceedings with no corresponding full article published in a peer-reviewed journal or no specific data provided even after contacting the author were excluded.

#### Participants

The studies involved participants aged above 18 years, who were diagnosed with knee OA by a physician or self-reported a physician diagnosis along with matching items based on the American College of Rheumatology clinical criteria [40,41], and had not undergone knee arthroplasty.

#### Interventions

Studies that were included in the review compared the effects of internet-based rehabilitation programs with conventional rehabilitation (eg, rehabilitation performed in the clinic or hospital) or waiting without any therapy. Internet-based rehabilitation could be the only intervention or could be combined with another form of physiotherapy. The internet-based rehabilitation programs were performed through videos or graphic knowledge demonstrations, real-time communication with physicians or therapists, and group discussions to promote the self-rehabilitation for individuals with knee OA. Rehabilitation methods include exercise, patient education, and self-management. Interventions used for participants had to be internet-based such as by email, websites, or software systems. Studies using noninternet technology support or not explicitly stating that internet technology was used to support the intervention were excluded, such as telephone, DVD, and cable television.

#### Outcome Measures

The main outcome measures were focused on pain (eg, the Western Ontario and McMaster [WOMAC] pain subscale, visual analog scale [VAS], Numerical Pain Rating Scale [NPRS]) and physical function (eg, WOMAC functional subscale, 30-second chair stand test, Timed Up and Go Test [TUG], and Knee Injury and OA Outcome Score [KOOS] functional subscale) for patients with knee OA. The primary outcome scale or the most representative scale was selected for analysis if multiple scales were used to evaluate the same outcome index in a study.

### Search Methods for Identification of Studies

Two authors (LW and LQW) independently reviewed the search results and screened the titles, abstracts, and full texts of identified references to select potentially eligible studies, which were imported into EndNote X8 (Clarivate Analytics, Philadelphia, PA, USA).

### Data Extraction and Management

Two authors (LW and LQW) completed data extraction independently and assessed the risk of bias for the included studies. A final decision was made after discussion with authors QW and CH in cases of any disagreement related to the data extraction process. Finally, the data were summarized in a previously standardized worksheet of Excel for Windows 2010. When the reported data were insufficient, we contacted the authors for more information.

The extracted data included: basic information of the study (eg, first author, year of publication, country, email address of the corresponding author); risk of bias (based on the PEDro scale) [42]; participants (overall sample size and sample size for each condition, overall mean age and the mean age for each condition, and the number of men and women); type of intervention for the experimental group (name of the program, components of the program, intervention time, delivery location); type of intervention for the control group (same as above); and outcomes (eg, the WOMAC pain and functional subscale, VAS, TUG). Outcomes reported as continuous variables are presented as the mean (SD).

### Quality Assessment

Quality assessments were performed with the PEDro scale [42]. The PEDro tool is based on the Delphi List criteria, which was used to evaluate the methodological quality in this study, and is considered to be valid and reliable [42-44]. All included trial reports were checked in the PEDro database to confirm their PEDro scale score. Considering that criterion 1 was not utilized to calculate the score, the sum of the other criteria could have a maximum of 10 points. Trials with a score ≥6 points were classified as “good,” whereas those with a score ≤5 points were graded as “poor” [45]. The poor-quality studies were excluded from the analysis. The quality of studies was assessed by two authors (SX and KS) using the PEDro scale and associated notes on administration of the PEDro scale [46] independently if a score was not available in the PEDro database [47]. Any dispute was settled through discussion or with consultation of a third reviewer (QW).

### Statistical Analysis

The mean (SD) of continuous outcome variables after therapy was used to calculate the total effect size via the mean difference and 95% CI. The standardized mean difference (SMD) was calculated when studies used different methods or scales to measure the same outcome. We assessed heterogeneity visually and based on the I² statistic [48]. The forest plots for the meta-analysis are presented along with a description of the results. A random-effects model was applied when substantial heterogeneity was observed (*P*<.05 or I^2^>50%); otherwise, a fixed-effects model was used [49]. Review Manager version 5.3 (Cochrane Collaboration, Copenhagen, Denmark) was employed for the statistical analyses and to produce forest plots.

## Results

### Search and Selection

**Figure 1:**
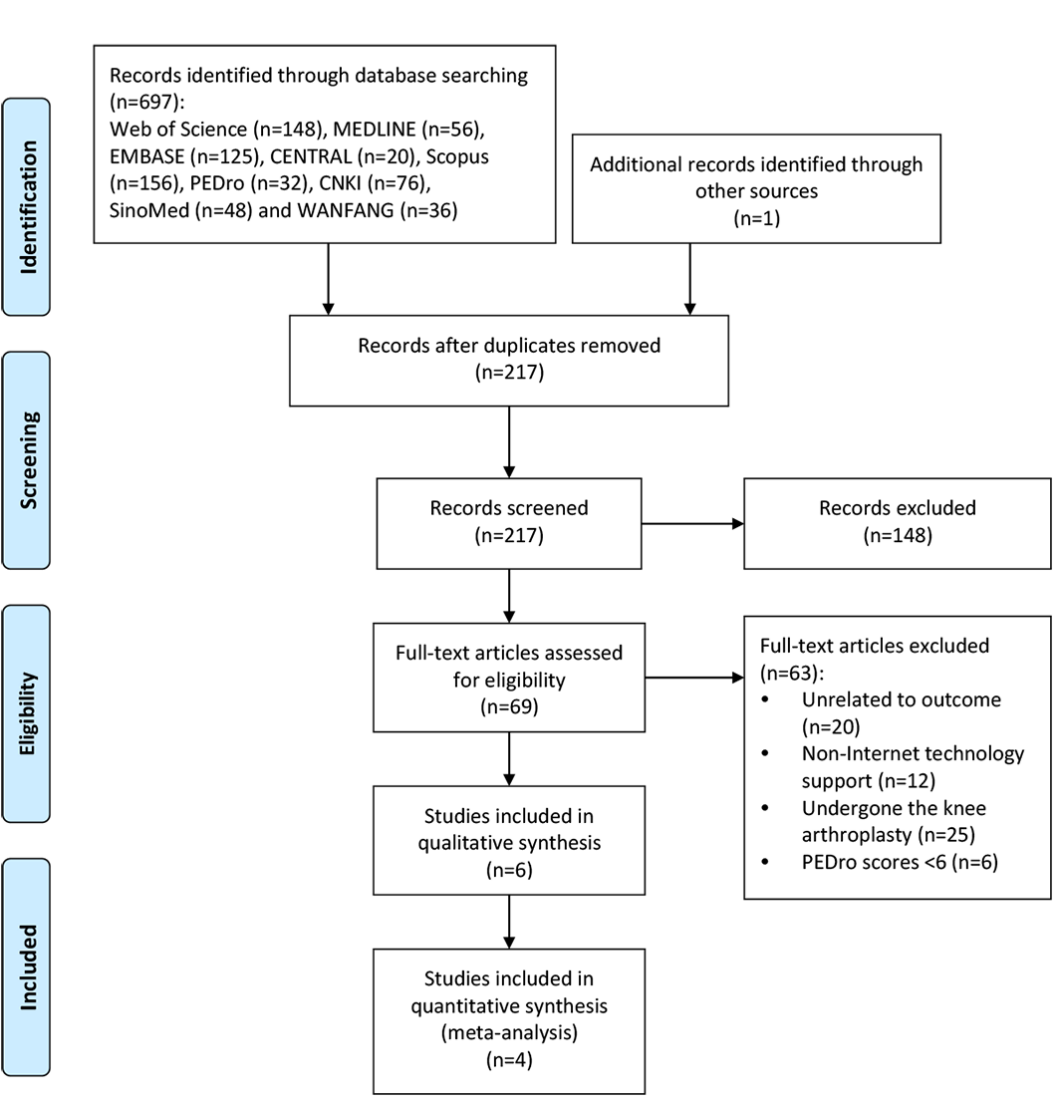
PRISMA (Preferred Reporting Items for Systematic reviews and Meta-Analyses) flow diagram. CENTRAL: Cochrane Central Register of Controlled Trials; EMBASE: Excerpta Medica Database; MEDLINE: Medical Literature Analysis and Retrieval System Online; PEDro: Physiotherapy Evidence Database; CNKI: China National Knowledge Infrastructure.

A total of 697 publications were retrieved through electronic searching from the databases. After exclusion of the duplicated studies and irrelevant subjects via the initial screening of titles and abstracts, 12 articles were systematically reviewed with 6 studies further excluded due to low quality based on a PEDro score ≤5 points. The list of eligible studies was sent to experts in the field to confirm that no other studies could be identified. In addition, the final articles included in the systematic review and meta-analysis were determined according to the guidelines of Cochrane Handbook for Systematic Reviews of Interventions [50]. Finally, 6 studies were identified for the systematic review, 4 of which were included in the meta-analysis, involving a total of 791 patients with knee OA (Figure 1).

### Study Characteristics

The baseline descriptive characteristics (country, sample size, age, and gender) of the 6 studies included in the systematic review are summarized in Table 1. Two studies were from the United States [35,51], one from Australia [33], one from China [34], one from Brazil [52], and one from the Netherlands [53]. The mean age of patients with knee OA ranged from 53.1 (SD 8.5) to 72.25 (SD 8.84) years, and all studies included both men and women.

**Table 1:** Baseline descriptive characteristics of studies included in the systematic review.

Reference, year, country	Patient characteristics		Comparison	Intervention	Intervention time (weeks)	Outcome
	N	Age (years), mean (SD)				
Aily et al [52]^a^, 2020, Brazil	20 (10 women, 10 men)	Group 1 (n=10): 54.8 (8.3); Group 2 (n=10): 53.1 (8.5)	Group 1: supervised periodized circuit training with load progression, 3 times a week	Group 2: same exercise protocol as group 1, but orientations to the exercises provided through videos on a website, DVD, or YouTube	14	VAS^b^, WOMAC^c^, 30-s chair stand test, 40-m fast-paced walk test, stair climb test
Huang et al [34]^a^, 2019, China	40 (30 women, 10 men)	Group 1 (n=20): 72.25 (8.84); Group 2 (n=20): 67.25 (10.97)	Group 1: conventional rehabilitation in the clinic	Group 2: conventional rehabilitation plus a brief GOH^d^-based intervention (educational lectures, medical suggestions, and psychotherapy)	24	WOMAC
O’Moore [33]^a^, 2018, Australia	69 (55 women, 14 men)	Group 1 (n=25): 59.68 (6.01); Group 2 (n=44): 63.16 (7.38)	Group 1: treatment as usual.	Group 2: iCBT^e^ program for depression added to treatment as usual	10	ASES^f^, WOMAC
Allen et al [35]^a^, 2018, United States	350 (251 women, 99 men)	Group 1 (n=140): 65.7 (10.3); Group 2 (n=68): 64.3 (12.2); Group 3 (n=142): 65.3 (11.5)	Group 1: physiotherapy (evidence-based approach); Group 2: wait without any therapy	Group 3: internet-based exercise training	48	WOMAC, 30-s chair stand, TUG^g^, 2-min step test, unilateral stand time
Rini et al [51], 2015, United States	113 (91 women, 22 men)	Group 1 (n=55): 66.67 (11.02); Group 2 (n=58): 68.52 (7.65)	Group 1: wait without any therapy	Group 2: PainCOACH program through the internet	8-10	AIMS2^h^ 5-item arthritis pain subscale, ASES, AIMS2 subscales relevant to lower extremity functioning, 20-item PASS^i^, AIMS2 comorbidities subscale
Bossen et al [53], 2013, Netherlands	199 (129 women, 70 men)	Group 1 (n=99): 63.0 (5.4); Group 2 (n=100): 61.0 (5.9)	Group 1: waitlist control without any therapy	Group 2: Join2move, a fully automated web-based intervention without human support	48	Self-reported PA^j^ (PASE^k^ and ActiGraph GT3X triaxial accelerometers), KOOS^l^ (functional subscale), self-perceived effect, NRS^m^, ASES

^a^Included in the meta-analysis.

^b^WOMAC: Western Ontario and McMaster Universities Osteoarthritis index.

^c^VAS: visual analog scale.

^d^GOH: Guangdong Online Hospital.

^e^iCBT: internet cognitive behavior therapy.

^f^ASES: Arthritis Self Efficacy Scale.

^g^TUG: Timed Up and Go Test.

^h^AIMS2: Arthritis Impact Measurement Scale 2.

^i^PASS: Pain Anxiety Symptoms Scale.

^j^PA: physical activity.

^k^PASE: Physical Activity Scale for the Elderly.

^l^KOOS: Knee Injury and Osteoarthritis Outcome Score.

^m^NRS: numeric rating scale.

### Intervention Programs

The internet-based rehabilitation programs used in the included RCTs are summarized in Table 1. Various internet-based rehabilitation programs have been developed in the included studies. To promote physical fitness, Allen et al [35] from the United States developed an internet-based exercise training program (IBET) containing tailored exercises, exercise progression, video demonstrations, automated reminders, and guidance on progression for patients with OA. Participants were encouraged to complete strengthening and stretching exercises at least 3 times per week and to engage in aerobic exercises daily [35]. Similarly, Rini et al [51] from the United States developed the PainCOACH program, which is a web-based platform that offers physical, psychological, and occupational therapies. PainCOACH includes 8 modules related to cognitive or behavioral pain coping skills in a self-directed manner (eg, without therapist contact) at a frequency of one per week. Each module took 35 to 45 minutes to complete [51]. Huang and colleagues [34] from China developed an internet-based rehabilitation program for patients with knee OA comprised of three broad segments: encouragement, educational lectures, and medical issues, each of which could be completed within 20-30 min in an independent manner. In Australia, O’Moore and colleagues [33] studied the effectiveness of an internet-based cognitive-behavioral therapy (iCBT) program for older adults with knee OA. The iCBT Sadness Program consists of six online lessons assigned as regular homework and provides access to supplementary resources. Aily and colleagues [52] from Brazil allowed patients with knee OA to utilize a website or YouTube videos for rehabilitation at home 3 times a week. They also provided periodic telephone calls to motivate, clarify, and monitor the performance of patients. In addition, a behavior-graded activity program named Join2move was developed by Bossen et al [53] in the Netherlands with the aim of promoting the self-management of behaviors of patients with knee OA when they are at home or in the community setting. The intervention period ranged from 8-10 weeks [51] to 48 weeks [35,53] (Table 1).

### Risk of Bias

All 6 studies included in the review scored greater than 6 points on the PEDro scale (Table 2). Even though the greatest risk of bias lies in the nonblinding of participants, in general, the total score of the 6 studies showed high methodological rigor [54,55] despite the fact that all studies had inadequate blinding of participants and therapists, and 2 studies had inadequate blinding of outcome assessors. One study did not provide adequate follow up. Two studies did not perform an intention-to-treat analysis. Overall, the methodological quality of the included studies was assessed as “good.”

**Table 2:** Assessment of methodological quality using the PEDro scale.

Quality metric	Aily et al [52]	Huang et al [34]	O’Moore et al [33]	Allen et al [35]	Rini et al [51]	Bossen et al [53]
Eligibility criteria	Yes	Yes	Yes	Yes	Yes	Yes
Random allocation	Yes	Yes	Yes	Yes	Yes	Yes
Concealed allocation	Yes	Yes	Yes	Yes	Yes	Yes
Baseline comparability	Yes	Yes	Yes	Yes	Yes	Yes
Blinded subjects	No	No	No	No	No	No
Blinded therapists	No	No	No	No	No	No
Blinded assessors	No	Yes	Yes	Yes	Yes	No
Adequate follow up	Yes	Yes	Yes	Yes	Yes	No
Intention-to-treat analysis	No	No	Yes	Yes	Yes	Yes
Between-group comparisons	Yes	Yes	Yes	Yes	Yes	Yes
Point estimates and variability	Yes	Yes	Yes	Yes	Yes	Yes
Total score^a^	6	7	8	8	8	6
Quality assessment	Good	Good	Good	Good	Good	Good

^a^Eligibility criteria did not contribute to the total score: 1=yes, 0=no.

### Outcomes of Interest

#### Pain

Pain is the most disabling symptom for patients with knee OA [5]. Six studies included in the review evaluated the effect of internet-based rehabilitation on osteoarthritic pain. The WOMAC pain subscale was used in 4 studies [33-35,52], with scores on the pain subscale ranging from 0 (no dysfunction) to 20 (maximum dysfunction) based on a 5-point Likert format, or ranging from 0 (no dysfunction) to 50 (maximum dysfunction) with the 11-box numerical rating scale format [56]. In the study of Aily et al [52], the WOMAC and VAS were both used to assess pain. The VAS is a 100-mm line and the participants are required to place a mark between the left side (0, representing “no pain”) and the right side (100, representing “the worst pain imaginable”). The 10-NPRS and the Arthritis Impact Measurement Scale 2 (AIMS2) were used in the other two studies, respectively [51,53]. The NPRS is scored in a similar manner to the VAS, except that the NPRS is scored from 0 to 10 (0 means no pain and 10 means the worst possible pain). AIMS2 is comprised of a 5-item arthritis pain subscale indicating the severity of arthritis pain (1 means severe and 5 means none) and the frequency of severe pain [57]. A study that did not apply the WOMAC function subscale measures showed a significant improvement in patients with knee OA after 3 months of internet-based rehabilitation based on the NPRS, but no significant change was observed after 12 months compared with the control group [53]. In another study using the AIMS2 pain subscale, the pain after 8-10 weeks of internet-based rehabilitation was found to be significantly improved in women but not in men compared with that of the control group [51].

**Figure 2:**
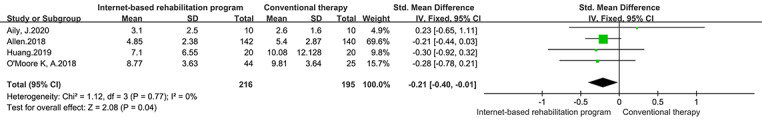
Forest plot of included studies comparing the effect of the internet-based intervention and conventional rehabilitation on pain according to the Western Ontario and McMaster Universities Osteoarthritis (WOMAC) pain subscale.

The meta-analysis on the effect of internet-based rehabilitation on osteoarthritic pain as measured by the WOMAC pain subscale contained 4 independent studies, involving a total of 411 participants [33-35,52]. We did not find evidence of significant heterogeneity among these studies (I^2^=0%, *P*=.77); therefore, a fixed-effects model was used. The meta-analysis showed that internet-based rehabilitation could significantly reduce the pain of patients with knee OA compared with conventional rehabilitation as assessed by the WOMAC pain subscale (SMD –0.21, 95% CI  −0.4 to –0.01, *P*=.04; Figure 2).

#### Physical Function

Improving patients’ functional conditions is the objective of rehabilitation for patients with knee OA. Physical function was assessed in 4 studies using the WOMAC function subscale [33-35,52]. The total score of the WOMAC function subscale (17 items) ranges from 0 (no dysfunction) to 68 (maximum dysfunction) with a 5-point Likert response format or from 0 (no dysfunction) to 170 (maximum dysfunction) with the 11-box numerical rating scale format [56]. One study assessed physical function using the KOOS function subscale [53]. The KOOS is a self-administered questionnaire to assess functional status regarding the patient’s knee problems on a 5-point Likert scale [58]. In another study, the AIMS2 subscales were used to evaluate the function of the lower extremities [51]. The TUG, 40-meter fast-paced walk test, 2-minute step test, unilateral stand time, and 30-second chair stand test were used to assess physical function for patients with knee OA in two studies [35,52]. The studies that did not apply the WOMAC function subscale measures showed that internet-based rehabilitation could not significantly improve physical function compared with the control group [35,52], even compared with the waitlist group as a control [35]. Only one study suggested that the physical function could be significantly improved after 3 months of internet-based rehabilitation compared with the waitlist group. However, the beneficial effect did not last after 12 months [53].

**Figure 3:**
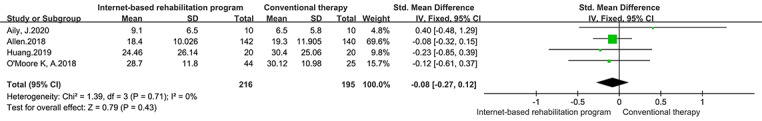
Forest plot of included studies comparing the effect of the internet-based intervention and conventional rehabilitation on physical function based on the Western Ontario and McMaster Universities Osteoarthritis (WOMAC) function subscale.

The 4 studies included in the meta-analysis on the effect of internet-based rehabilitation on function as measured by the WOMAC function subscale involved a total of 411 participants [33-35,52]. The fixed-effects model was used in this analysis owing to the low heterogeneity (I^2^=0%, *P*=.71). The results indicated that internet-based rehabilitation could not significantly improve the physical function of patients with knee OA compared with the control group according to the WOMAC function subscale (SMD –0.08, 95% CI −0.27 to 0.12, *P*=.43; Figure 3). 

## Discussion

### Principal Findings

This systematic review and meta-analysis investigated whether internet-based rehabilitation programs could effectively improve the pain and physical function in patients with knee OA. The findings showed that internet-based rehabilitation could significantly improve the pain of patients with knee OA but not the physical function. Qualitative synthesis was performed for 6 studies and the meta-analysis was performed for 4 studies, comprising a total of 791 patients with knee OA. These trials showed good methodological quality as assessed by high PEDro scores (>6). However, only a very small number of studies could be included in the review.

Knee OA often causes pain, which is a significant reason for patients to be admitted to the hospital. This meta-analysis showed that internet-based rehabilitation could assist patients with knee OA to self-manage and even relieve their pain after they are discharged from the hospital. The programs such as IBET and the web-based intervention Join2move were demonstrated to be effective for pain reduction in patients with knee OA [35,53]. The IBET program focused on exercise interventions tailored to patients’ needs, and played a role in pain control [35]. Similarly, Join2move, developed by Bossen and colleagues [53], adopts a behavior-graded activity program to assist patients with OA to gradually increase their daily activities in a fixed amount of time [53]. These internet-based rehabilitation programs could combine various interventions based on the patient’s behavioral, psychological, family, and social factors, which can be carried out at home or in the community setting to alleviate osteoarthritic pain.

Furthermore, knee OA could lead to the decline of patients’ physical function such as walking, shopping, and housework [59]. This meta-analysis showed that internet-based rehabilitation could not significantly improve the physical function of patients with knee OA compared with conventional rehabilitation. Allen et al [35] reported that patients who underwent 12 months of internet-based strengthening and stretching exercises at least 3 times per week did not obtain a significant improvement of physical function compared with those who received conventional rehabilitation through face-to-face supervised exercise. Even though the patients’ physical function could be improved after 3 months of the Join2move internet-based rehabilitation, the positive effects were not detectable at follow up of 12 months compared with the waitlist group [53]. It was postulated that the undetectable improvement after internet-based rehabilitation might be due to the fact that the recruited participants often had better baseline physical function and could accomplish the tasks assigned by the programs [60,61]. Alternatively, we also speculate that functional improvement may require a longer-term intervention and more intervention forms that can integrate daily life factors and improve lifestyle function, rather than simply the external exercise components. These results are comparable with the results of other meta-analyses [62,63]. Wang et al [62] showed that a telerehabilitation program (eg, telephone counseling/coaching, video conferencing) could be effective for pain control but not for functional improvement in patients after knee OA replacement surgery. Another systematic review also showed that the improvements in physical function were not significant for patients with knee OA through telerehabilitation exercise compared with either control or waitlist groups [63].

It is critical for the designers or health care providers to develop appropriate modules comprised in the internet-based rehabilitation programs. In this systematic review, we found that the programs for knee OA possessed some common modules such as exercise guidance, psychological intervention, knee OA education, and cognitive behavior management. In addition, the different programs were manifested through the specific modules. For example, the PainCOACH program focused on the behavioral and cognitive management of pain control for patients with knee OA, whereas the Join2move program aimed to enhance the physical function [51,53]. In the future, comprehensive and personalized modules will need to be developed to achieve the integration of facilities and patients in the community or home setting, and to monitor the safety and progress via wearable devices when performing exercises or behavior management for patients with knee OA. The modules designed for each individual can be personalized based on big data analysis collected from the wearable devices.

### Limitations

There are several limitations to this study. First, there were only 6 studies that could be included in the systematic review and only 4 studies that were eligible to be included in the meta-analysis. Thus, more high-quality RCTs with larger sample sizes in this field are needed. Second, the included participants were mainly patients with knee OA who had not undergone arthroplasty or other surgical interventions. Third, the outcome measures used to assess pain and physical function included in the studies of internet-based rehabilitation were subjective. Fourth, analyses of moderator variables on the effects of the internet-based rehabilitation programs (eg, age, gender, sample size) were not performed. Fifth, considering the diversity of outcome indicators and the small number of included studies, only the studies using the WOMAC scale were included in the meta-analysis to ensure the reliability of the study and comparison. We plan to update this systematic review and meta-analysis with the increase of research on internet-based rehabilitation for knee OA in the future. Finally, only 2 studies included a follow-up period of 12 months, indicating a lack of assessments on the long-term effects of internet-based rehabilitation. 

### Conclusion

Internet-based rehabilitation is a promising strategy for patients with knee OA to obtain access to rehabilitation guidance and monitoring at home or in the community setting. The results of this systematic review and meta-analysis indicate that internet-based rehabilitation programs involving personalized modules could improve the pain but not the physical function of patients with knee OA compared with conventional rehabilitation. More high-quality studies with large samples are needed, with a focus on the long-term outcomes of internet-based rehabilitation for patients with knee OA.

## Appendix

Multimedia Appendix 1 Search strategy.

## Abbreviations

AIMS2: Arthritis Impact Measurement Scale 2
IBET: internet-based exercise training
iCBT: internet cognitive behavior therapy
KOOS: Knee Injury and Osteoarthritis Outcome Score
NPRS: Numeric Pain Rating Scale
OA: osteoarthritis
PEDro: Physiotherapy Evidence Database
RCT: randomized controlled trial
SMD: standardized mean difference
TUG: The Timed Up and Go Test
VAS: The visual analog scale
WOMAC: The Western Ontario and McMaster Universities Osteoarthritis
