# Supplementary material for: Effect of Internet-Based Rehabilitation Programs on Improvement of Pain and Physical Function in Patients with Knee Osteoarthritis: Systematic Review and Meta-analysis of Randomized Controlled Trials
Source: J Med Internet Res. 2021 Jan 5;23(1):e21542. doi: 10.2196/21542 (PMC7815452; doi:10.2196/21542)
Supplement: Multimedia Appendix 1 [file jmir_v23i1e21542_app1.docx]

Multimedia Appendix 1: Search Strategy

Database: Ovid MEDLINE(R) <1946 to 2020>

Date searched: 1 May 2020

Search Strategy:

--------------------------------------------------------------------------------

1 exp Telemedicine/ or exp Telerehabilitation/ or exp E-health/ or exp Telehealth/ (27878)

2 exp Internet/ or exp Internet-Based Intervention/ or exp Web/ or exp Online/ or exp App/ or exp Wearable/ or exp Sensor/ (78248)

3 exp Osteoarthritis, Knee/ (19649)

4 1 or 2 (102295)

5 3 and 4 (56)

Database: EBM Reviews - Cochrane Central Register of Controlled Trials <May 2020>

Date searched: 1 May 2020

Search Strategy:

--------------------------------------------------------------------------------

1 exp Telemedicine/ or exp Telerehabilitation/ or exp E-health/ or exp Telehealth/ (2304)

2 exp Internet/ or exp Web/ or exp Online/ or exp App/ or exp Wearable/ or exp Sensor/ (3854)

3 exp Rehabilitation/ (23709)

4 2 and 3 (512)

5 1 or 4 (2711)

6 exp Osteoarthritis, Knee/ (4112)

7 5 and 6 (10)

8 2 and 6 (18)

9 7 or 8 (20)

Database: Embase <1974 to 2020 >

Date searched: 1 May 2020

Search Strategy:

--------------------------------------------------------------------------------

1 telemedicine/ or telerehabilitation/ or internet rehabilitation/ or web/ or online/ or app/ or wearable/ or sensor/ (129175)

2 exp telehealth/ (44198)

3 exp knee osteoarthritis/ or knee osteoarthrosis/ or knee cartilage/ or degenerative arthritis (32357)

4 1 or 2 (147476)

5 3 and 4 (125)

Database: China national knowledge infrastructure (CNKI)

Date searched: 1 May 2020

Search Strategy:

--------------------------------------------------------------------------------

1 SU='远程康复' OR SU='远程医疗' OR SU='互联网' OR SU='微信' OR SU='QQ'(377590)

2 SU='膝骨关节炎' OR SU='关节炎' OR SU='膝关节炎' (95764)

3 1 and 2 (76)

Database: WANFANG Data

Date searched: 1 May 2020

Search Strategy:

--------------------------------------------------------------------------------

1 (( "远程康复"[常用字段:智能] OR "远程医疗"[常用字段:智能] OR "互联网"[常用字段:智能] OR "移动医疗"[常用字段:智能] OR "微信"[常用字段:智能] OR "QQ"[常用字段:智能] OR "手机"[常用字段:智能])) (26876)

2 (( "膝关节炎"[常用字段:智能] OR "膝骨关节炎"[常用字段:智能] OR "关节炎"[常用字段:智能])) (112209)

3 1 and 2 (36)
